# Effectiveness of high-power laser use in the removal of esthetic restorations: a systematic review

**DOI:** 10.1007/s10103-026-05037-6

**Published:** 2026-09-15

**Authors:** Daniele das Graças Silva, ⁠⁠Nayra Kamylle Araujo Fernandes Carvalho, Diêgo de Oliveira Camargos, Arthur Henrique Vieira de Almeida, Valder Ferreira da Silva Filho, Paula Cristina Pelli Paiva, Olga Dumont Flecha, Brender Leonan-Silva

**Affiliations:** 1 Dentistry school, Universidade Federal dos Vales do Jequitinhonha e Mucuri, Diamantina, Brazil; 2 Postgraduate Program in Dentistry, Federal University of the Jequitinhonha and Mucuri Valleys (UFVJM), Diamantina, Minas Gerais 39100000 Brasil; 3 Dentistry school, Faculdade Sete Lagoas (FACSETE), Sete Lagoas, Brazil

**Keywords:** Laser therapy, Dental restoration repair, Composite resins, Treatment outcome

## Abstract

This systematic review aimed to evaluate the effectiveness of high-power lasers in the removal of esthetic restorations compared with conventional methods. The review was conducted in accordance with the Preferred Reporting Items for Systematic Reviews and Meta-Analyses (PRISMA) and registered in PROSPERO (registration number: CRD420251055736). A comprehensive electronic search was performed in MEDLINE, Scopus, Embase, Web of Science, and the Cochrane Library. Study selection, data extraction, and methodological quality assessment were independently performed by four reviewers using QUIN tool for in vitro studies. A total of 2.384 records were identified, of which 19 in vitro studies conducted on extracted human permanent teeth met the eligibility criteria and were included in the qualitative synthesis. The included studies evaluated different high-power laser systems, predominantly Er: YAG and Er, Cr: YSGG lasers, as well as CO₂ lasers. Five studies were classified as high quality, whereas the remaining fourteen studies were classified as moderate quality. Available laboratory evidence suggests that laser-assisted techniques may represent a potential alternative approach for removing esthetic restorations, with advantages including conservative tissue removal, reduced damage to underlying tooth structures, and intrapulpal temperature increases within acceptable limits. The Er: YAG laser was the most frequently investigated system, with power settings ranging from 1.5 to 5.9 W, showing particularly favorable laboratorial performance in the removal of lithium disilicate restorations, especially in thinner substrates. Within the limitations of the available evidence, high-power lasers appear to be a promising and minimally invasive alternative to conventional methods for the removal of esthetic restorations. However, the predominance of in vitro studies highlights the need for well-designed clinical trials to confirm these findings and support their clinical application.

**Supplementary Information:**

The online version contains supplementary material available at https://doi.org/10.1007/s10103-026-05037-6.

## Introduction

Contemporary dentistry has increasingly prioritized esthetic and minimally invasive procedures, aiming to preserve the remaining tooth structure while achieving optimal therapeutic outcomes [1, 2]. In this context, the development of new restorative materials and techniques has expanded the possibilities for reproducing the natural appearance of teeth [2]. Among the most widely used materials are resin-based composites, which are employed for their favorable esthetic and functional properties, and their affordable cost [3]. In addition, ceramic materials such as lithium disilicate and zirconia are frequently used in prosthetic procedures because of their biocompatibility, chemical stability, and excellent esthetic performance [4, 5].

In clinical practice, the removal of esthetic restorations is relatively common. However, the removal of restorative materials such as composite resins or ceramics remains a challenge for clinicians, particularly with respect to preserving tooth structure. Although conventional methods, such as the use of diamond or carbide burs in high-speed handpieces, are effective in reducing clinical time, they present important limitations related to the selectivity of substrate removal and may lead to a considerable increase in intrapulpal temperature [6]. Therefore, techniques that allow the removal of these materials without damaging, or at least minimizing damage to, the underlying tooth structure have been increasingly investigated in conservative dentistry [7].

The use of high-power laser therapy (HPLT) has been investigated for removing restorative materials. This includes the Er: YAG laser, Er, Cr: YSGG laser, Cr: YSGG laser, and CO₂ laser, with power settings from 1.5 to 10 W [6, 7]. These systems may offer advantages, such as selective ablation of restorative materials. This helps protect health dental structures. Thermal safety can also be maintained by keeping intrapulpal temperature below critical levels, and then could otherwise compromise pulpal health. Additionally, lasers have shown efficient performance with satisfactory clinical removal times [7].

Despite the high survival rates of contemporary esthetic restorations, replacement remains a common clinical procedure. Restoration removal may be required because of secondary caries, marginal degradation, debonding, fracture or chipping of the restorative material, esthetic dissatisfaction, color mismatch, loss of retention, endodontic treatment, or the need to access the underlying tooth structure for retreatment. Conventional removal with rotary instruments is often time-consuming, non-selective, and may result in unnecessary loss of sound dental tissue or damage to the restoration, particularly when retrieval rather than destruction is desired [1, 6, 7]. Therefore, conservative techniques that facilitate restoration removal while preserving both the restoration and the underlying tooth structure have become an important area of investigation.

Although several in vitro studies have investigated the use of high-power lasers for the removal of esthetic restorations, the available evidence remains fragmented because of differences in laser systems, restorative materials, and experimental protocols. Moreover, previous reviews have primarily focused on specific laser systems or particular restorative materials. To the best of our knowledge, no previous systematic review has comprehensively synthesized the available evidence on high-power laser systems used for the removal of esthetic restorations across different restorative materials and laboratory outcomes. Therefore, the present systematic review aimed to critically evaluate the effectiveness, safety, and methodological characteristics of high-power laser-assisted removal of esthetic restorations based on the current in vitro evidence.

## Materials and methods

### Protocol registration

This systematic review was conducted in accordance with the Preferred Reporting Items for Systematic Reviews and Meta-Analyses (PRISMA) guidelines and was prospectively registered in PROSPERO (CRD420251055736, on 20th May, 2025) before the literature search was initiated, and no deviations from the registered protocol occurred during the conduct of this systematic review.

### Eligibility criteria

The search strategy was developed based on the PICOS framework, considering the following components: Population (extracted human permanent teeth restored with esthetic restorative materials), Intervention (removal of restorations using high-power lasers, including the Er: YAG laser, Er, Cr: YSGG laser, and CO₂ laser), Comparator (conventional mechanical removal methods or different laser parameters, restorative materials, or experimental conditions when applicable. Studies without a comparator were also considered eligible if they evaluated the effectiveness or safety of high-power laser-assisted removal), Outcomes (O): The primary outcome was removal/debonding time of esthetic restorations. Secondary outcomes included intrapulpal temperature variation, bond strength, structural damage, enamel and dentin preservation, and surface alterations associated with laser-assisted procedures, and Study design (in vitro studies conducted on extracted human permanent teeth). Based on these criteria, the following research question was formulated: “What is the effectiveness and thermal safety of high-power laser systems in the removal of esthetic restorations, including their impact on restoration debonding, thermal changes, and structural integrity?”.

To address this question, all included studies were in vitro experiments conducted on extracted human permanent teeth. Eligible studies were required to evaluate the removal of esthetic restorations using any type of HPLT. No language restrictions were applied during the search process. Studies published in any language were considered for eligibility. Additionally, narrative reviews, systematic or scoping reviews, letters, book chapters, conference abstracts, opinion articles, and observational studies were excluded. Studies without available full text after institutional search and/or attempts to contact the corresponding authors were excluded.

### Search strategy

An electronic literature search strategy was carried out in MEDLINE, Scopus, Web of Science, Cochrane Library, and Embase, on March 19th, 2026, and updated on July, 2026. The search strategy was initially developed and applied in MEDLINE using indexed MeSH terms and their synonyms. Minor refinements were subsequently made to adapt the strategy to the specific characteristics of the other databases. The complete search strategies for all electronic databases are provided in Table 1. No searches of gray literature sources were performed.

**Table 1:** Search strategy for all databases

Databases	Search strategy Date: March 19, 2026 updated July, 2026	Studies found
Medline (PubMed)	(laser* OR “Er: YAG” OR “Er, Cr: YSGG”) AND (“Dental Restoration” OR “Composite Resin” OR “Ceramic Veneer” OR “Esthetic Restoration”) AND (remov* OR debond* OR elimination)	213
Web of Science	((laser* OR “Er: YAG” OR “Er, Cr: YSGG”) AND (“Dental Restoration” OR “Composite Resin” OR “Ceramic Veneer” OR “Esthetic Restoration” OR zirconia OR “lithium disilicate”) AND (remov* OR debond* OR retriev*))	426
Scopus	TITLE-ABS-KEY ((laser* OR “Er: YAG” OR “Er, Cr: YSGG”) AND (“Dental Restoration” OR “Composite Resin” OR “Ceramic Veneer” OR “Esthetic Restoration” OR veneer* OR zirconia OR “lithium disilicate” OR “ceramic crown”) AND (remov* OR debond* OR retriev*))	720
Cochrane Library	(laser* OR “Er: YAG” OR “Er, Cr: YSGG”) AND (“Dental Restoration” OR “Composite Resin” OR “Ceramic Veneer” OR “Esthetic Restoration” OR veneer* OR zirconia OR “lithium disilicate” OR “ceramic crown”) AND (remov* OR debond* OR retriev*)	57
Embase	(‘laser’/exp OR laser*:ti, ab OR ‘er: yag’:ti, ab OR ‘er, cr: ysgg’:ti, ab) AND (‘dental restoration’/exp OR ‘composite resin’/exp OR ‘ceramic veneer’:ti, ab OR ‘esthetic restoration’:ti, ab OR veneer*:ti, ab OR zirconia: ti, ab OR ‘lithium disilicate’:ti, ab OR ‘ceramic crown’:ti, ab) AND (remov*:ti, ab OR debond*:ti, ab OR retriev*:ti, ab)	968
Total		2.384

### Study selection

After the electronic search, the retrieved references were exported to the systematic review management platform Rayyan (https://new.rayyan.ai/). Duplicates were identified using Rayyan software and subsequently manually verified by two independent reviewers to ensure accuracy before study selection. Prior to the screening process, the reviewers conducted a calibration exercise to standardize the application of the eligibility criteria. Disagreements were discussed until consensus was reached before the formal screening process. Three reviewers were involved in the article selection process to minimize any potential selection bias. In the first phase, two reviewers (AHVA and DOC) independently and blindly screened the titles and abstracts. The articles were coded as “preselected” for those that met the inclusion criteria, “possibly” for potentially eligible/unclear records, or “excluded”. Divergences of opinion were resolved by discussion. When this was not possible, a third reviewer (DDGS) was consulted to reach a consensus. In the second phase, the same two reviewers applied the eligibility criteria during the full-text analysis of the preselected articles. Divergences of opinion were resolved by discussion. When this was not possible, a third reviewer (DDGS) was consulted and the impasse was resolved through a consensus meeting among the reviewers.

### Data extraction

Prior to data extraction, the standardized extraction form was pilot-tested using a sample of included studies and refined where necessary to ensure consistency and completeness. Two reviewers (AHVA and DOC) independently extracted relevant data from the full-text articles onto a customized data extraction sheet. After the data extraction process, the two reviewers performed the cross-checking of the information. For each paper, the following data were systematically extracted: publication details (author, year, country, study design), sample sizes, interventions, assessment criteria, main results and conclusion.

In cases of missing or incomplete data, attempts were made to contact the corresponding authors of the included studies for clarification. When data could not be obtained, the available information was analyzed as reported in the original publications, and missing data were not imputed.

### Data synthesis

Data were synthesized using a narrative approach due to the substantial heterogeneity among included studies in terms of laser parameters, restorative materials, study designs, and outcome measures.

Studies were grouped according to restorative material (veneers, crowns, composite restorations, and mixed experimental models) and laser system (Er: YAG, Er, Cr: YSGG, and CO₂ lasers).

A meta-analysis was not performed due to variability in study designs, outcome definitions, and measurement methods, which precluded meaningful statistical pooling of results. Instead, a qualitative synthesis was conducted to identify patterns across studies.

### Heterogeneity domains

The following domains of heterogeneity were predefined and considered for qualitative synthesis: laser type (Er: YAG, Er, Cr: YSGG, CO₂), wavelength, power output, pulse frequency, pulse duration, cooling or irrigation protocol, restorative material (zirconia, lithium disilicate, feldspathic ceramic, composite resin), restoration thickness, cement type, and outcome measures (e.g., debonding time, bond strength, intrapulpal temperature variation, and structural alterations).

### Quality assessment and risk of bias

Four reviewers (AHVA, DOC, DDGS, and NKAFC) independently and in duplicate assessed the methodological quality and risk of bias of the included studies using the Quality Assessment Tool for In Vitro Studies (QUIN Tool), developed and validated by Sheth et al. [8].

The QUIN tool comprises 12 methodological domains specifically designed for in vitro dental research, including: clearly stated aims/objectives, sample size calculation, sampling technique, comparison group, methodology, operator details, randomization, outcome measurement, outcome assessor details, blinding, statistical analysis, and presentation of results. Each item was scored as 2 (adequately specified), 1 (inadequately specified), or 0 (not specified). Items considered not applicable were excluded from the final calculation, according to the original QUIN methodology.

An overall quality score was calculated for each study by dividing the total score obtained by the maximum possible score and expressing the result as a percentage. Studies were classified as high quality (≥ 70%), moderate quality (50–69%), or low quality (< 50%). Disagreements between reviewers were resolved through discussion until consensus was reached. A detailed description of all QUIN domains and scoring criteria is provided in Supplementary Table S1.

## Results

### Study selection

**Fig. 1:**
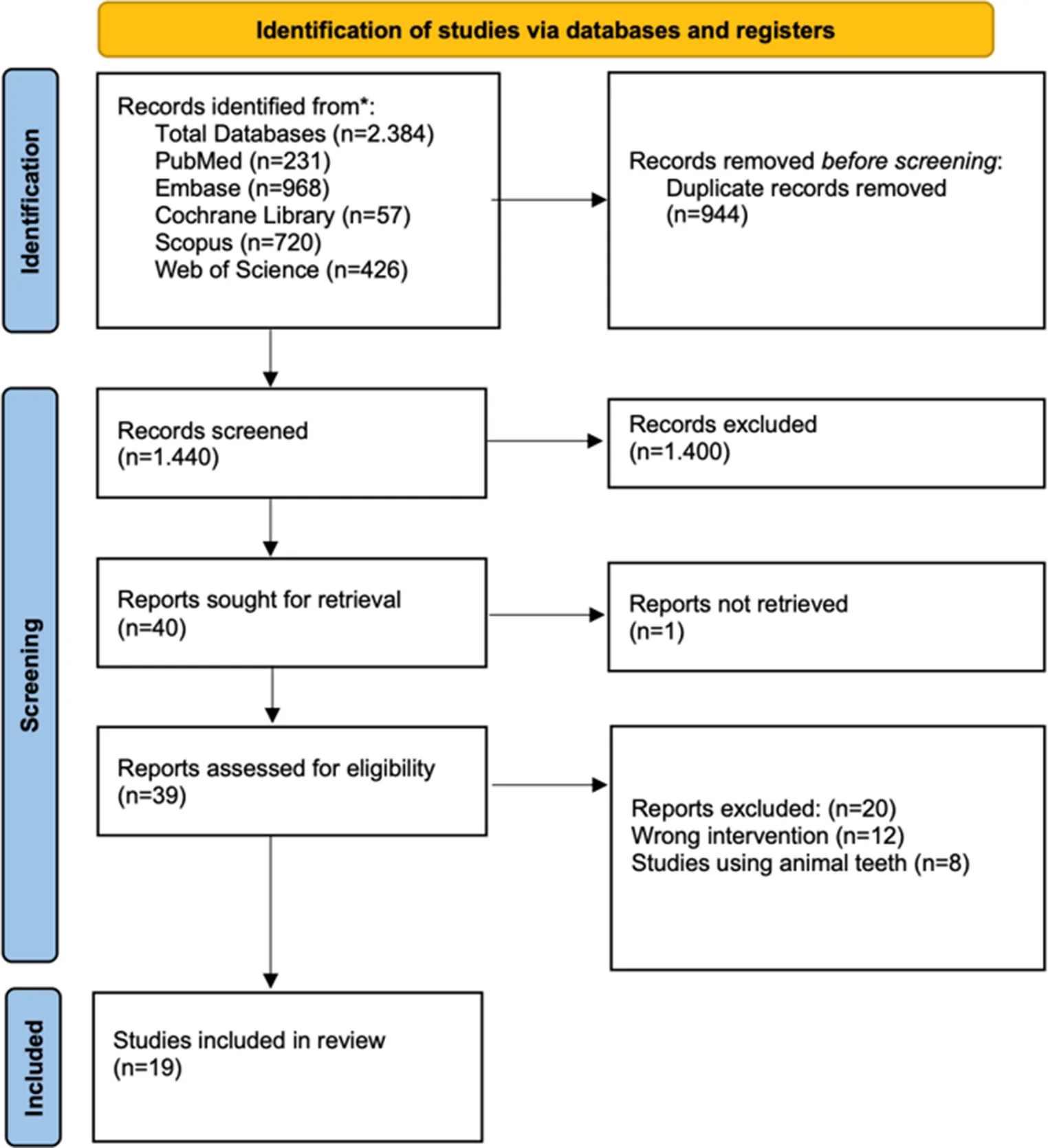
Flowchart of the study selection process and the reasons for exclusion

The initial search strategy retrieved 2.384 records across all databases. A total of 944 duplicate records were identified and removed prior to screening; 1.440 records remained and were screened based on titles and abstracts (phase 1). Forty articles were then assessed through full-text reading; of these, one was excluded due to the unavailability of the full text. At the end of the selection process, 19 in vitro studies conducted on extracted human permanent teeth were included (Fig. 1).

### Study characteristics

The included articles were conducted across four continents, encompassing seven countries. Asia and North America accounted for the largest number of publications, each contributing five studies. In North America, four studies were conducted in the United States and one in Canada. In Asia, the three studies originated from Turkey, one from Iraq and the United Arab Emirates. Europe contributed two studies, both from Switzerland. Africa was represented by one study conducted in Egypt. No studies were identified from South America or Oceania. Sample sizes ranged from 22 to 120 teeth, including premolars, incisors, and molars.

#### Laser systems used and materials evaluated

Er: YAG and Er, Cr: YSGG lasers were the most frequently investigated systems, while one study evaluated a CO₂ laser. Considerable heterogeneity was observed regarding wavelength, power output, pulse duration, pulse frequency, irradiation time, and cooling parameters. The evaluated laser powers ranged from 1.5 W to 7.5 W, depending on the restorative material and clinical purpose.

The included studies evaluated different restorative materials, including lithium disilicate, zirconia, feldspathic ceramics, leucite-reinforced ceramics, nanoceramic resin, and composite resin restorations. Several studies also investigated the influence of restoration thickness and cement type on laser-assisted removal of esthetic restorations performance.

The most frequently assessed outcomes were debonding time, shear bond strength, intrapulpal temperature variation, and structural integrity after laser irradiation. Additional outcomes included surface morphology assessed by scanning electron microscopy, adhesive remnant index, enamel preservation, and thermal behavior during irradiation.

Overall, laser-assisted removal of esthetic restorations was considered effective and conservative across most studies. Lithium disilicate restorations generally demonstrated shorter debonding times compared with zirconia and leucite-based ceramics. Higher laser power settings tended to improve debonding efficiency but, in some cases, resulted in greater thermal variation. Most studies reported intrapulpal temperature increases within clinically acceptable limits and minimal structural damage to restorations or dental tissues.

Comparator methods included conventional mechanical removal using high-speed diamond burs, no-laser control groups, and comparisons among different laser parameters and restorative materials.

### Results of individual sources of evidence

The interventions focused on evaluating the efficiency, safety, and thermal effects of laser irradiation for the removal or debonding of dental restorations under different experimental conditions. The Er: YAG laser was the most frequently applied intervention, being used in several studies with different power settings and experimental conditions. The Er, Cr: YSGG laser was also widely employed, particularly for debonding ceramic restorations and veneers. One study additionally evaluated the use of a CO₂ laser with a spectral feedback system for the selective removal of composite resin. A comprehensive description of the characteristics of the studies included in the present review is provided in Tables 2, 3, 4 and 5.

**Table 2:** Data synthesis of veneers (including occlusal veneers)

Author (year)	Sample sizes	Laser system	Comparator	Outcomes assessed	Main findings
AlBalkhi, et al. (2018) [9]	40 extracted human maxillary premolars restored with lithium disilicate veneers (5 groups; *n* = 8)	Er: YAG laser (2940 nm; contact and non-contact modes; 3.0–5.4 W)	Contact vs. non-contact modes and different energy/frequency settings	Debonding time, intrapulpal temperature, failure mode	Er: YAG successfully debonded all veneers. Non-contact mode reduced debonding time but caused greater temperature increases, remaining within safe limits. Laser parameters influenced efficiency and thermal changes; predominant failure occurred within the resin cement.
Yilmaz et al. (2019) [10]	120 human maxillary central incisors restored with IPS Empress II ceramic discs (0.5, 1.0, and 2.0 mm; *n* = 10/group)	Er, Cr: YSGG laser	Different ceramic thicknesses, cement types (Panavia F vs. RelyX ARC), and laser vs. no-laser application	Shear bond strength, failure pattern (SEM), Adhesive Remnant Index (ARI)	Laser application enabled complete debonding of 0.5 mm ceramics without fractures. Bond strength decreased after laser exposure, particularly with thinner ceramics.
Walinski et al. (2021) [11]	22 human central incisors restored with CAD/CAM leucite-reinforced ceramic veneers (IPS Empress CAD; 0.75 ± 0.03 mm)	Er, Cr: YSGG laser (333 mJ/pulse; 30 Hz)	No control/comparator group; laser-assisted debonding evaluation	Debonding time and intrapulpal temperature variation	Er, Cr: YSGG laser enabled effective veneer debonding with intrapulpal temperature maintained within safe limits.
AlBalkhi; Hamadah (2022) [12]	46 extracted non-carious human maxillary premolars restored with 0.7-mm lithium disilicate veneers (IPS e.max LT; 36 specimens allocated after pilot study)	Er: YAG laser (2940 nm; 270 mJ/pulse; 15 Hz; 4.05 W; non-contact mode)	Different pulse durations (SSP, MSP, SP) and water/air cooling ratios (1:1 vs. 3:3)	Debonding time, intrapulpal temperature variation, and failure mode	All laser protocols successfully debonded veneers. Shorter pulse durations (SSP and MSP) showed faster and safer debonding, with temperature increases remaining below the critical threshold.
Amin; Jamal (2022) [13]	30 extracted maxillary first premolars restored with lithium disilicate veneers (IPS e.max Press; 0.5 and 1.0 mm; 2 groups)	Er, Cr: YSGG laser	Comparison between 0.5 mm and 1.0 mm lithium disilicate veneer thicknesses	Debonding time and failure pattern	Veneer thickness influenced laser-assisted removal time. Failures were mainly adhesive, with residual cement remaining on both tooth and ceramic surfaces.
Zhu et al. (2023) [14]	33 extracted human teeth restored with occlusal veneers (Vita Mark II, Vita Amber, and resin ceramic; 1.0, 1.5, and 2.0 mm thicknesses)	Er: YAG laser (2.5–3.5 W)	Different ceramic materials and veneer thicknesses	Debonding time, SEM analysis, and surface alterations	Higher laser power reduced debonding time, whereas increased ceramic thickness prolonged removal. Resin ceramic veneers showed limited debonding effectiveness. Minimal enamel alterations were observed after laser application.
El-Damanhoury et al. (2022) [15]	48 extracted human maxillary central incisors restored with lithium disilicate veneers (IPS e.max Press)	Er: YAG laser (1.5–5.4 W)	Different veneer thicknesses (0.5 vs. 1.0 mm) and Er: YAG power settings	Debonding time and intrapulpal temperature variation	Thinner veneers showed faster debonding. Higher power improved removal efficiency for thicker veneers but increased thermal variation. All protocols enabled safe debonding without structural damage.
Amin. (2023) [16]	45 maxillary premolars restored with veneers of: FC, CAD/CAM LD, and pressed LD (ingot), divided into three groups	Er, Cr: YSGG laser	Comparison among feldspathic ceramic, CAD/CAM lithium disilicate, and pressed lithium disilicate veneers	Veneer debonding time by ceramic type	Efficient veneer removal in all groups. Lithium disilicate showed shorter debonding time. No enamel damage observed
Halloum et al. (2025) [17]	20 extracted human maxillary central incisors restored with lithium disilicate veneers (IPS e.max Press)	Er: YAG laser (2940 nm; 400 mJ; 10 Hz; MSP; non-contact)	Hydrofluoric acid surface treatment vs. Er: YAG laser surface treatment before cementation	Debonding time and failure mode	Hydrofluoric acid-treated veneers showed shorter debonding time compared with laser-conditioned veneers. Adhesive failure predominated in both groups, with no differences in failure pattern.
Raafat et al. (2025) [18]	63 extracted human maxillary central incisors restored with 0.7-mm lithium disilicate veneers (IPS e.max CAD; 9 experimental groups, *n* = 7; 1 unbonded control)	Er, Cr: YSGG laser (2780 nm; 15 Hz; 4–6 W; 1%, 20%, or 40% water)	Different laser power outputs and water percentages	Debonding time, intrapulpal temperature, translucency parameter, and SEM surface morphology	Higher power reduced debonding time but increased surface alterations. The 5 W/20% water protocol provided the best balance between efficiency, thermal safety, and preservation of ceramic properties.

*CP* Composite resin, *G* group, *CAD/CAM* Computer-Aided Design/Computer-Aided Manufacturing, *Z* Zirconia, *LD* Lithium disilicate, *FC* Feldspathic ceramic, *IV* In Vitro study, *MPa* Megapascal, *SEM* Scanning Electron Microscopy, *Er*, *Cr*: *YSGG* Erbium, Chromium: Yttrium Scandium Gallium Garnet, *Er*: *YAG* Erbium-doped Yttrium Aluminium Garnet

**Table 3:** Data synthesis of crowns

Author (year)	Sample sizes	Laser system	Comparator	Outcomes assessed	Main findings
Grzech-Leśniak et al. (2020) [19]	26 extracted human molars restored with lithium disilicate and zirconia crowns cemented with resin cement	Er: YAG laser	Comparison between lithium disilicate and zirconia crowns	Removal time, intrapulpal temperature, and structural alterations	Zirconia crowns required longer removal time and showed lower temperature increases than lithium disilicate crowns. Laser application maintained safe thermal conditions and did not cause damage to crowns or tooth structure.
Golob Deeb et al. (2022) [20]	31 extracted teeth (18 anterior primary teeth and 13 posterior permanent teeth) with prefabricated zirconia crowns	Er, Cr: YSGG laser (2780 nm; 5.0 W; 15 Hz; 50% water/50% air)	Conventional high-speed rotary handpiece with diamond bur	Crown removal time, intrapulpal temperature, and SEM evaluation of crown/tooth surfaces	Both methods successfully removed zirconia crowns. Rotary removal was faster, whereas laser application resulted in greater crown preservation and fewer dentin alterations, enabling potential crown reuse. Temperature increases with laser remained below harmful thresholds.
Gozneli et al. (2023) [21]	27 intact premolars prepared for lithium disilicate crowns (IPS e.max CAD; 3 groups according to thickness: 1.0, 1.5, and mixed thickness; *n* = 9/group)	Er: YAG laser (2940 nm; 5.0–5.9 W)	Different crown thicknesses and laser power settings	Crown debonding time and intrapulpal temperature variation	All laser protocols enabled crown removal. Higher power reduced debonding time but increased thermal risk, particularly in thinner crowns. Thicker crowns allowed efficient removal without structural damage.
Suliman et al. (2024) [22]	40 human premolars prepared for CAD/CAM crowns (4 groups; *n* = 10/group)	Er: YAG laser (335 mJ; 15 Hz; 5.0 W; 50 ms)	Zirconia vs. lithium disilicate crowns and resin cements (Panavia V5 vs. RelyX Ultimate)	Crown debonding time, ceramic type, cement system, and SEM interface analysis	Er: YAG laser enabled safe and effective debonding of both zirconia and lithium disilicate crowns. No differences in removal time were observed between cement systems.

*CP* Composite resin, *G* group, *CAD/CAM* Computer-Aided Design/Computer-Aided Manufacturing, *Z* Zirconia, *LD* Lithium disilicate, *FC* Feldspathic ceramic, *IV* In Vitro study, *MPa* Megapascal, *SEM* Scanning Electron Microscopy, *Er*, *Cr*: *YSGG* Erbium, Chromium: Yttrium Scandium Gallium Garnet, *Er*: *YAG* Erbium-doped Yttrium Aluminium Garnet

**Table 4:** Data synthesis of composite restorations

Author (year)	Sample sizes	Laser system	Comparator	Outcomes assessed	Main findings
Babarasul et al. (2021) [23]	30 extracted human premolars	Er: YAG laser (20 Hz and 25 Hz)	High-speed diamond bur	Removal time, cavity alterations, and SEM surface morphology	Laser-assisted composite removal was more conservative and caused fewer surface alterations compared with diamond bur removal

*CP* Composite resin, *G* group, *CAD/CAM* Computer-Aided Design/Computer-Aided Manufacturing, *Z* Zirconia, *LD* Lithium disilicate, *FC* Feldspathic ceramic, *IV* In Vitro study, *MPa* Megapascal, *SEM* Scanning Electron Microscopy, *Er*, *Cr*: *YSGG* Erbium, Chromium: Yttrium Scandium Gallium Garnet, *Er*: *YAG* Erbium-doped Yttrium Aluminium Garnet

**Table 5:** Data synthesis of mixed/comparative studies

Author (year)	Sample sizes	Laser system	Comparator	Outcomes assessed	Main findings
Çulhaoğlu et al. (2021) [24]	120 maxillary incisors restored with CAD/CAM veneers (IPS e.max CAD, Vita CEREC Blocs, and Lava Ultimate; 0.5 and 1.0 mm thicknesses)	Er: YAG laser	No-laser control; comparison among restorative materials and thicknesses	Shear bond strength (SBS) and SEM surface morphology	Er: YAG laser enabled effective debonding without enamel damage or restoration fracture. Greater efficiency was observed for lithium disilicate and thinner restorations, allowing potential immediate recementation.
Eid et al. (2021) [25]	84 sound premolars	Er: YAG (2940 nm) and Er, Cr: YSGG (2870 nm) lasers	Er: YAG vs. Er, Cr: YSGG vs. no-laser control	Debonding efficiency, debonding time, and shear bond strength	Both laser systems were effective for glass ceramic debonding. Ceramic thickness influenced removal time, and lithium disilicate restorations showed faster debonding than leucite-based ceramics.
Luca et al. (2025) [26]	16 extracted human teeth with 32 restorations	Er: YAG laser (275 mJ; 20 Hz; 5.5 W; 10 mm distance; maximum 5 min irradiation)	Different ceramic materials: monolithic zirconia, layered zirconia, lithium disilicate, and feldspathic ceramic	Debonding success, debonding time, and restoration integrity	Overall debonding success was 71.9%. Successful removal was achieved for restorations ≤ 1.0 mm, with higher success for layered zirconia, feldspathic ceramic, and lithium disilicate. Monolithic zirconia showed no successful debonding. No enamel damage was observed.
Tharp et al. (2025) [27]	Maxillary premolars	Er, Cr: YSGG laser (7.5 W, 25 Hz)	Comparison among dentin, dentin analog (NEMA G10), and composite resin substrates	Thermal variation during laser irradiation	Dentin analog (NEMA G10) showed thermal behavior comparable to dentin. Maximum temperature increases remained within safe pulpal limits.

*CP* Composite resin, *G* group, *CAD/CAM* Computer-Aided Design/Computer-Aided Manufacturing, *Z* Zirconia, *LD* Lithium disilicate, *FC* Feldspathic ceramic, *IV* In Vitro study, *MPa *Megapascal, *SEM* Scanning Electron Microscopy, *Er*, *Cr*: *YSGG* Erbium, Chromium: Yttrium Scandium Gallium Garnet, *Er*: *YAG* Erbium-doped Yttrium Aluminium Garnet

Considerable methodological heterogeneity was observed among the included studies. Variations were identified regarding laser systems (Er, Er, Cr, and CO₂), irradiation parameters (power output, pulse frequency, pulse duration, wavelength, and irradiation time), restorative materials (zirconia, lithium disilicate, feldspathic ceramics, nanoceramic resin, and composite resin), restoration thicknesses, and experimental protocols. In addition, the evaluated outcomes differed substantially across studies, including debonding time, shear bond strength, intrapulpal temperature variation, surface morphology, adhesive remnant index, and structural integrity analyses. Differences in study design, specimen preparation, bonding procedures, and outcome assessment further contributed to the overall heterogeneity. Therefore, direct comparisons among studies were limited, and a quantitative synthesis (meta-analysis) was considered inappropriate.

A summary of methodological and clinical heterogeneity across included studies is presented in Table 6, including laser parameters, restorative materials, irradiation conditions, and outcome variability.

**Table 6:** Summary of methodological and clinical heterogeneity across included studies

Variable	Range/description (based on included studies)
Laser systems/wavelength	Er: YAG (2940 nm); Er, Cr: YSGG (2780–2790 nm range); CO₂ laser (9.3 μm)
Power output	1.5–7.5 W (Er: YAG/Er, Cr: YSGG studies); CO₂ laser reported in energy/frequency settings rather than W
Pulse frequency	15–30 Hz (most erbium laser studies); CO₂ laser 200 Hz
Pulse duration	10–60 ms where reported; not consistently reported across studies (NR in several studies)
Cooling/irrigation	Water spray cooling used in most Er: YAG and Er, Cr: YSGG studies; CO₂ laser study used spectral feedback system without conventional irrigation
Irradiation time	Approximately 10–60 s or until debonding occurred; several studies reported time-to-debonding instead of fixed irradiation time
Debonding/removal time	Approximately 3 s to > 120 s depending on material, thickness, and laser settings (veneers generally faster than crowns)
Thermal outcomes (intrapulpal temperature)	Generally within safe limits (< critical threshold) in most studies; transient increases reported especially at higher power settings

### Quality assessment and risk of bias

The methodological quality and risk of bias assessment performed using the QUIN tool demonstrated that the overall quality of the included studies ranged from 58.3% to 70.8% (Table 7.). Five studies were classified as high quality (≥ 70%), whereas the remaining fourteen studies were classified as moderate quality (50–69%). No study was classified as low quality.

**Table 7.**
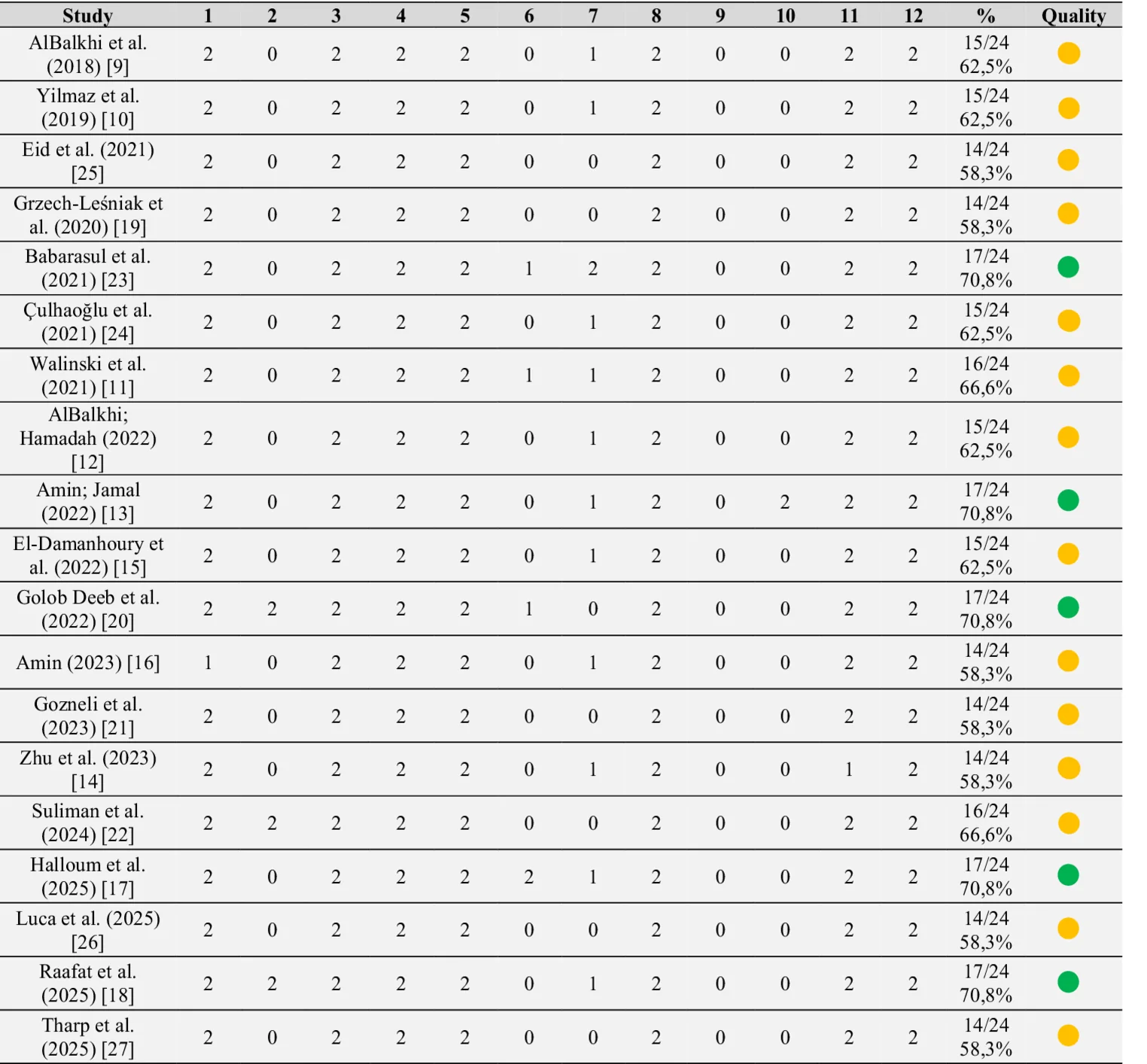
Methodological quality and risk-of-bias assessment of the included studies using the quality assessment tool for in vitro studies (QUIN)

Scoring according to the QUIN (Quality Assessment Tool for In Vitro Studies) proposed by Sheth et al. [8] Each domain was scored as follows: 2 = adequately specified; 1 = inadequately specified; 0 = not specified. Overall methodological quality was calculated as the percentage of the maximum obtainable score (24 points) and categorized as high (≥70%), moderate (50–69%), or low (<50%). Green indicates high methodological quality and yellow indicates moderate methodological quality.

Most studies clearly described their objectives, sampling procedures, comparison groups, experimental methodology, outcome measurements, statistical analyses, and presentation of results. However, important methodological limitations were consistently identified, particularly regarding sample size calculation, randomization, blinding, and reporting of outcome assessor details, which were absent or insufficiently described in most studies. Detailed scoring for each QUIN domain is presented in Supplementary Table S1.

## Discussion

The findings of this systematic review indicate that current in vitro evidence suggests that high-power laser therapy (HPLT), particularly Er: YAG and Er, Cr: YSGG systems, has the potential to facilitate the removal of esthetic restorations under laboratory conditions. Compared with conventional mechanical methods, the available laboratory evidence suggests that laser-assisted techniques may enable more controlled restoration removal and better preservation of dental structure. However, these observations are derived exclusively from in vitro studies and should not be interpreted as evidence of clinical superiority or effectiveness. Laser technology has been increasingly investigated in restorative dentistry due to its potential to support minimally invasive procedures and improve procedural precision, particularly in adhesive and debonding protocols [28]. Nevertheless, the substantial heterogeneity among studies prevents quantitative synthesis, and the findings should therefore be interpreted with caution.

A meta-analysis could not be performed because of substantial heterogeneity across the included studies. Clinical heterogeneity was evident in the diversity of laser systems (e.g., Er: YAG, Er, Cr: YSGG, CO₂), restorative materials (composite resin, lithium disilicate, feldspathic ceramics, leucite-reinforced ceramics, and zirconia), restoration thicknesses, and evaluated outcomes, including debonding time, bond strength, intrapulpal temperature, surface integrity, and enamel preservation. Methodological heterogeneity was also considerable, arising from differences in experimental designs, specimen preparation protocols, storage conditions, laser irradiation parameters (power, energy, pulse duration, frequency, irradiation time, cooling protocols, and tip-to-surface distance), comparator methods, and outcome assessment techniques. Owing to this marked clinical and methodological variability, statistical heterogeneity could not be meaningfully assessed, and pooling of the data was considered inappropriate. Consequently, a descriptive synthesis was conducted in accordance with the PRISMA Statement.

Most studies were classified as having moderate methodological quality according to the QUIN assessment, mainly due to insufficient reporting of sample size calculation, randomization, blinding, and outcome assessor details. These methodological limitations increase the potential risk of bias and should be considered when interpreting the available evidence.

Regarding thermal safety, intrapulpal temperature increase was generally maintained within acceptable biological limits when appropriate laser settings and cooling strategies were employed. Studies using Er, Cr: YSGG and Er: YAG lasers demonstrated that, although higher power settings may accelerate restoration removal, they can also increase thermal risk [9, 11, 12, 15, 21]. Importantly, continuous water spray irrigation was consistently identified as a critical factor in controlling heat generation. These findings are in agreement with previous evidence indicating that laser irradiation, when properly calibrated, does not induce harmful thermal effects on pulpal tissues [29]. The importance of controlling intrapulpal temperature is well established, as increases above critical thresholds may result in irreversible pulpal damage [30].

From a mechanistic perspective, the effectiveness of erbium lasers can be attributed to their high affinity for water and hydroxyapatite, enabling selective photo-thermal ablation of dental tissues and restorative materials. This selective interaction allows controlled removal with minimal damage to adjacent structures, which may explain the superior preservation of enamel and dentin compared with rotary instruments [31]. In addition to their affinity for water-containing substrates, the mechanism underlying laser-assisted debonding of ceramic restorations appears to involve transmission of laser energy through the ceramic material to the underlying resin cement layer. The absorbed energy may promote thermal softening, micro-explosions of residual water molecules, and degradation of the adhesive interface, resulting in reduced bond strength and facilitating restoration removal. The efficiency of this process is influenced by factors such as ceramic composition, thickness, optical properties, and laser parameters, which determine the amount of energy reaching the cement layer. This mechanism may explain the shorter debonding times frequently observed in thinner and more translucent ceramic restorations [10, 21, 23, 25].

Based on the available laboratory evidence, laser-assisted removal appears to offer potential advantages over conventional methods such as high-speed diamond burs. While rotary instruments are associated with non-selective wear and potential over-preparation, lasers may provide a more controlled and conservative approach under standardized laboratory conditions [23, 29]. This is particularly relevant in restorative dentistry, where preservation of sound tooth structure is a key clinical objective. Additionally, studies evaluating composite removal demonstrated that CO₂ lasers can achieve rapid and selective ablation with minimal damage to underlying enamel [27, 32]. Conventional rotary instruments, although widely used, are associated with non-selective removal of dental tissues and a higher risk of excessive tooth structure loss, reinforcing the clinical relevance of minimally invasive alternatives such as laser-assisted techniques [1].

Material-related factors, particularly thickness and composition, play a significant role in the effectiveness of laser-assisted removal. Overall, thinner restorations are removed more rapidly and with lower thermal risk, whereas increased thickness requires longer irradiation times and careful adjustment of laser parameters [11, 15, 25]. Evidence also suggests that complete debonding is more predictable in thinner ceramic materials [10, 13], and that bond strength may vary according to thickness and laser settings [19]. These findings reinforce the need for individualized parameter selection based on material characteristics.

The type of restorative material also influences laser performance. Lithium disilicate restorations appear to respond more favorably to laser-assisted removal, with shorter procedure times compared with feldspathic or leucite-reinforced ceramics [13, 15, 25]. In contrast, zirconia-based restorations tend to require longer irradiation times due to their lower absorption characteristics; however, removal remains feasible without significant adverse effects [22, 31].

Current in vitro evidence suggests that laser-assisted removal of esthetic restorations has the potential to become a conservative alternative for ceramic restoration debonding. Laboratory studies consistently reported preservation of the underlying tooth structure and reduced risk of iatrogenic damage under experimental conditions. However, these findings have not yet been confirmed in clinical settings [33, 34]. Er: YAG lasers have shown efficiency in debonding ceramic restorations by selectively targeting the adhesive layer, which contributes to reduced mechanical stress during removal [35].

This review presents several strengths, including transparent methodological procedures, clearly defined eligibility criteria, duplicate study selection and data extraction, and the inclusion of multiple electronic databases. Additionally, the findings were generally consistent across studies, supporting the potential of laser-based approaches under laboratory conditions. Although these findings provide valuable mechanistic information and may support the design of future clinical studies, they should not be interpreted as evidence for routine clinical application.

**Fig. 2:**
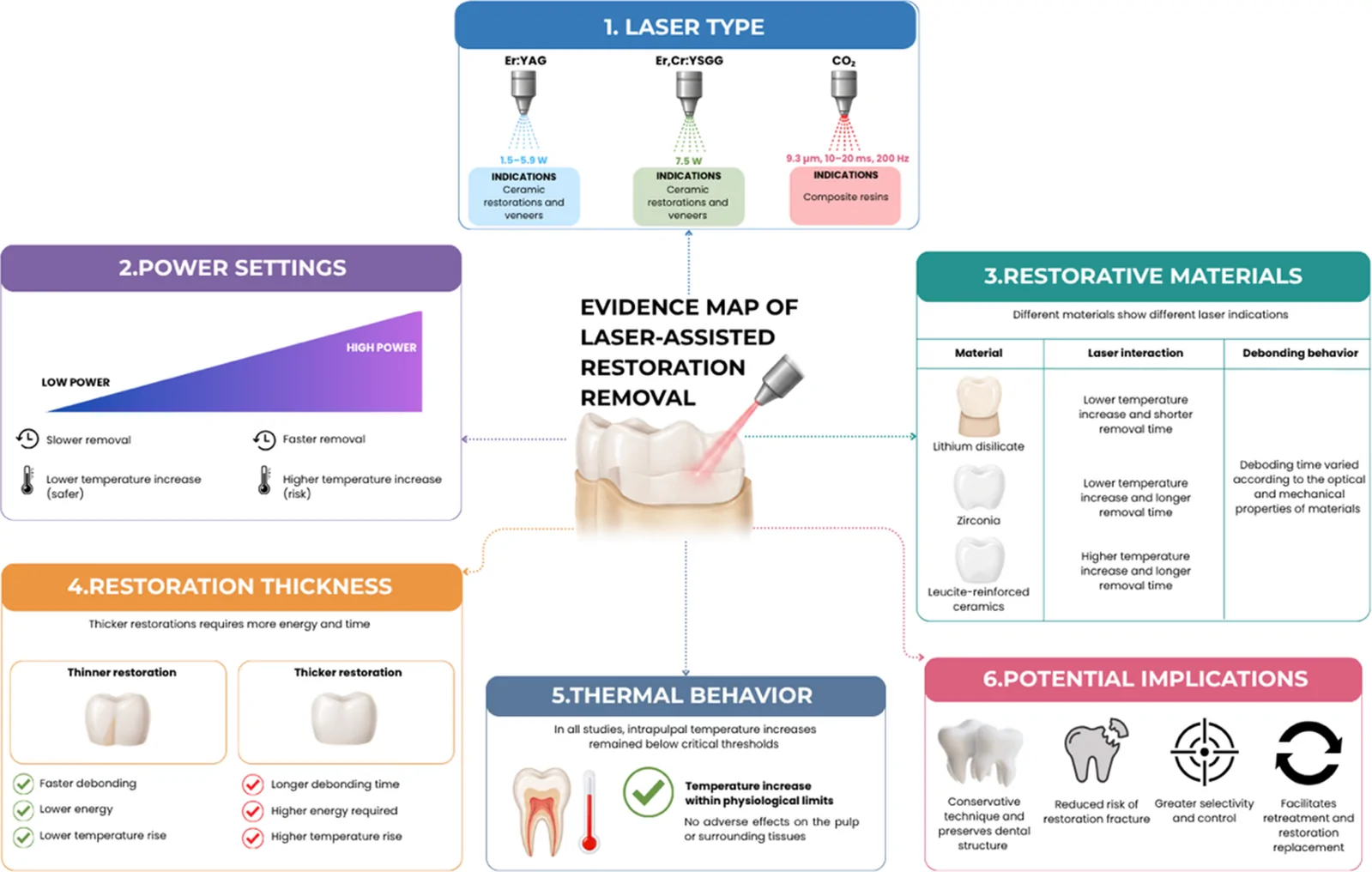
Overview of the evidence for laser-assisted restoration removal

When stratified by laser type, erbium-based lasers (Er: YAG and Er, Cr: YSGG) consistently demonstrated favorable laboratory performance in facilitating the debonding of ceramic restorations. Variations in wavelength and irradiation parameters influenced debonding efficiency, while restoration composition and thickness also affected removal time. Lithium disilicate restorations generally exhibited faster debonding than zirconia and leucite-reinforced ceramics, likely because of differences in optical properties and laser energy absorption. Across studies, intrapulpal temperature increases remained below commonly accepted biological thresholds under the investigated experimental conditions; however, biological safety under clinical conditions remains to be established. For a comprehensive overview of the available evidence according to laser system, restorative material, and reported outcomes, refer to Fig. 2.

Although current laboratory evidence suggests that laser-assisted removal may preserve dental structure and facilitate selective debonding, these findings cannot yet be extrapolated to clinical practice. Clinical implementation also remains limited by equipment costs, operator training, variability in laser protocols, and the lack of standardized clinical parameters.

The findings of the present review are consistent with previous systematic reviews reporting that erbium lasers have shown promising laboratory performance for debonding ceramic restorations while preserving dental structure and maintaining intrapulpal temperature within clinically acceptable limits. Previous evidence has also suggested that lithium disilicate restorations tend to exhibit shorter debonding times compared with zirconia-based restorations, likely due to differences in optical and thermal properties [36–38]. Unlike previous reviews that focused primarily on specific ceramic systems or individual laser types, the present review provides a broader synthesis of different laser systems and restorative materials, allowing a more comprehensive understanding of laser-assisted removal of esthetic restorations strategies in restorative dentistry.

Several limitations should be acknowledged. All included studies were conducted in vitro; therefore, the findings should be interpreted as preclinical evidence rather than evidence of clinical effectiveness. Laboratory models cannot reproduce important clinical variables such as saliva contamination, restoration aging, occlusal loading, patient-related variability, or operator-related factors. Furthermore, considerable heterogeneity in restorative materials, laser parameters, specimen preparation, and evaluated outcomes limited direct comparison among studies and precluded quantitative synthesis. The predominance of studies with moderate methodological quality further reduces confidence in the available evidence. Although the consistency of laboratory findings supports continued investigation of laser-assisted restoration removal, well-designed randomized clinical trials using standardized protocols and long-term follow-up are required before definitive clinical recommendations can be established.

## Conclusion

Current evidence from in vitro studies suggests that high-power lasers, particularly Er: YAG and Er, Cr: YSGG systems, may facilitate the removal of esthetic restorations, with the most consistent findings reported for ceramic restorations, including lithium disilicate and zirconia. However, the available evidence remains exclusively laboratory-based and is characterized by substantial methodological heterogeneity and predominantly moderate methodological quality, limiting the certainty and clinical applicability of the findings. Although intrapulpal temperature increases generally remained within acceptable limits under experimental conditions, these results should not be interpreted as evidence of clinical effectiveness or superiority over conventional techniques. Well-designed clinical studies with standardized protocols are required before laser-assisted removal of esthetic restorations can be recommended for routine clinical practice.

## Supplementary Information

Below is the link to the electronic supplementary material.

Supplementary File 1 (DOCX 16.2 KB)

## Data Availability

No datasets were generated or analysed during the current study.

## Abbreviations

HPLT: High-power laser therapy
Er:YAG: Erbium-doped Yttrium Aluminum Garnet
Er,Cr:YSGG: Erbium, Chromium-doped Yttrium Scandium Gallium Garnet
Cr:YSGG: Chromium-doped Yttrium Scandium Gallium Garnet
CO₂: Carbon Dioxide
PRISMA: Preferred Reporting Items for Systematic Reviews and Meta-Analyses
PROSPERO: International Prospective Register of Systematic Reviews
QUIN Tool: Quality Assessment Tool for In Vitro Studies
CP: Composite resin
G: group
CAD/CAM: Computer-Aided Design/Computer-Aided Manufacturing
Z: Zirconia
LD: Lithium disilicate
FC: Feldspathic ceramic
IV: In Vitro study
MPa: Megapascal
SEM: Scanning Electron Microscopy
