# Supplementary material for: Effectiveness of high-power laser use in the removal of esthetic restorations: a systematic review
Source: Lasers Med Sci. 2026 Sep 15;41(1):234. doi: 10.1007/s10103-026-05037-6 (PMC13578092; doi:10.1007/s10103-026-05037-6)
Supplement: Supplementary file 1 [file 10103_2026_5037_MOESM1_ESM.docx]

| **Supplementary Table S1. Scoring sheet for QUIN Tool (quality assessment tool for in vitro studies)** | | | | | |
| --- | --- | --- | --- | --- | --- |
| **Criteria No.** | **Criteria** | **Adequately Specified**  **(Score=2)** | **Inadequately Specified**  **(Score=1)** | **Not Specified**  **(Score=0)** | **Not**  **Applicable** |
| 1 | Clearly stated aims/objectives |  |  |  |  |
| 2 | Detailed explanation of sample size Calculation |  |  |  |  |
| 3 | Detailed explanation of sampling technique |  |  |  |  |
| 4 | Details of comparison group |  |  |  |  |
| 5 | Detailed explanation of methodology |  |  |  |  |
| 6 | Operator details |  |  |  |  |
| 7 | Randomization |  |  |  |  |
| 8 | Method of measurement of outcome |  |  |  |  |
| 9 | Outcome assessor details |  |  |  |  |
| 10 | Blinding |  |  |  |  |
| 11 | Statistical analysis |  |  |  |  |
| 12 | Presentation of results |  |  |  |  |
| Developed and validated by Sheth et al. (2024) [8] | | | | | |
